# High Purity and Yield of Boron Nitride Nanotubes Using Amorphous Boron and a Nozzle-Type Reactor

**DOI:** 10.3390/ma7085789

**Published:** 2014-08-11

**Authors:** Jaewoo Kim, Duckbong Seo, Jeseung Yoo, Wanseop Jeong, Young-Soo Seo, Jaeyong Kim

**Affiliations:** 1 Nuclear Materials Research Division, Korea Atomic Energy Research Institute (KAERI), 111-989 Daeduck-daero, Yuseong-gu, Daejeon-si 305-353, Korea; E-Mail: jsyoo@kaeri.re.kr; 2 World Class Institute (WCI) Quantum Beam based Radiation Research Center, Korea Atomic Energy Research Institute, 111-989 Daeduck-daero, Yuseong-gu, Daejeon-si 305-353, Korea; 3 Nuclear Technology Division, EnesG, 8 Techno10-ro, Yuseong-gu, Daejeon-si 305-510, Korea; E-Mail: duckbong.seo@enesg.co.kr; 4 Department of Nano Science and Technology, Sejong University, 98 Gunja-dong, Gwangjin-gu 143-747, Korea; 5 Department of Physics, Hanyang University, 222 Wangsimliro, Sungdong-gu, Seoul 133-791, Korea; E-Mails: mildpooh@hanmail.net (W.J.); kimjy@hanyang.ac.kr (J.K.)

**Keywords:** boron nitride nanotubes (BNNTs), amorphous boron, ball milling, annealing, core-shell structure, seed nanoparticles

## Abstract

Enhancement of the production yield of boron nitride nanotubes (BNNTs) with high purity was achieved using an amorphous boron-based precursor and a nozzle-type reactor. Use of a mixture of amorphous boron and Fe decreases the milling time for the preparation of the precursor for BNNTs synthesis, as well as the Fe impurity contained in the B/Fe interdiffused precursor nanoparticles by using a simple purification process. We also explored a nozzle-type reactor that increased the production yield of BNNTs compared to a conventional flow-through reactor. By using a nozzle-type reactor with amorphous boron-based precursor, the weight of the BNNTs sample after annealing was increased as much as 2.5-times with much less impurities compared to the case for the flow-through reactor with the crystalline boron-based precursor. Under the same experimental conditions, the yield and quantity of BNNTs were estimated as much as ~70% and ~1.15 g/batch for the former, while they are ~54% and 0.78 g/batch for the latter.

## 1. Introduction

Boron nitride nanotubes (BNNTs) are useful for various research and engineering applications, including electric insulating thermal conducting composites [1,2,3], ceramic composites having superplasticity [4,5,6] or metal composites [7], medical applications, such as drug delivery [8,9], boron neutron capture therapy [10], hydrogen storage [11,12,13], ultraviolet (UV) light emission [14], self-cleaning agents [15], radiation shielding [16,17], *etc.* However, the production of BNNTs has not reached the commercial stage yet, due to a lack of large-scale production; furthermore, the generation of impurities might be inevitable, requiring costly purification steps [18,19]. Among currently available synthetic methods for BNNTs, a ball milling-annealing process [20,21,22] appears to be more advantageous than other processes, such as arc discharge [23,24], chemical vapor deposition [25,26,27] and laser-based synthesis [28,29], because it uses a simple apparatus, a relatively low process temperature and a simple routine process that may enhance the production rate. However, the ball milling-annealing process still needs to be improved to achieve a commercial level of production; that is, to improve the low process efficiency, *i.e.*, the relatively long milling and annealing time, the generation of particulate impurities, relatively low yields, *etc.* Recently, high yield productions of BNNTs using ball milling and annealing were reported [30,31,32,33]. Most reports, however, are unable to present the specific quantities and yields of the BNNTs synthesis.

We have reported that the initial shape of the precursor nanoparticles produced from the ball milling of crystalline boron is important to determine the synthesis of boron nitride (BN) and the shape of BNNTs if they are cylinders or bamboo [22]. Furthermore, the crystallographic structure of boron coated on the surface of the catalytic precursor nanoparticles produced from the milling of crystalline boron was found to be a key factor for BNNTs synthesis [22,34]. Upon these fundamental findings, we explored the B/Fe interdiffused precursor nanoparticles produced from the ball milling of a mixture of amorphous boron, which with Fe can reduce the Fe impurity, while increasing the production yield of BNNTs compared to the crystalline boron-based precursor. In this investigation, we also introduce a nozzle-type reactor for BNNTs synthesis. A longer and more homogeneous exposure of the reactive nitrogenous gas to the precursor nanoparticles was possible by using this system and enhances the synthesis of BN and the growth of nanotubes (NTs) compared to a flow-through cylinder-type reactor. The yields of BNNTs were evaluated using the weight increase after annealing of the precursor nanoparticles, as well as the specific surface areas based on the BET measurements. We found that an amorphous B/Fe precursor was able to reduce the milling time, as well as the Fe impurity greatly in the BNNTs samples by using a simple purification process, and a nozzle-type reactor with the amorphous boron-based precursor increased the BNNTs production compared to the conventional flow-through reactors [20,21,22]. The method and system for the BNNTs synthesis presented in this investigation may offer a clue for large production and lead to a commercial stage.

## 2. Experimental Section

As a precursor for the synthesis of BNNTs, a mixture of amorphous boron powder (Novacentrix, Austin, TX, USA, 1 μm, purity >95%) and Fe powder (OCI Ltd., Seoul, Korea, ~100 μm, >99.0%) as a catalytic agent was milled using a planetary mill (P-100, Taemeong Scientific, Anyang, Korea). The amorphous boron powder contains several impurities, such as Mg > ~3.5%, and traces of Fe, O, N, *etc.* The stainless steel (STS) milling vessels and balls with a diameter of 5 mm were used for milling. Milling vessels were filled with 2 atm of N_2_ gas, and the ball-to-powder weight ratio was set at 100:4.4. The powder mixture consists of 4 g of amorphous boron and 0.4 g of Fe and was ball milled under 600 rpm for 6 h. The temperature of the milling system was maintained at room temperature or lower by flowing tap water. Ball-milled powder was dispersed in ethanol, and the supernatant was collected after the magnet was used to separate the large Fe particles. The collected particles from the supernatant were then annealed at 1200 °C under a flowing gas mixture of N_2_ (90 vol%) and NH_3_ (10 vol%) for 6 h.

**Figure 1:**
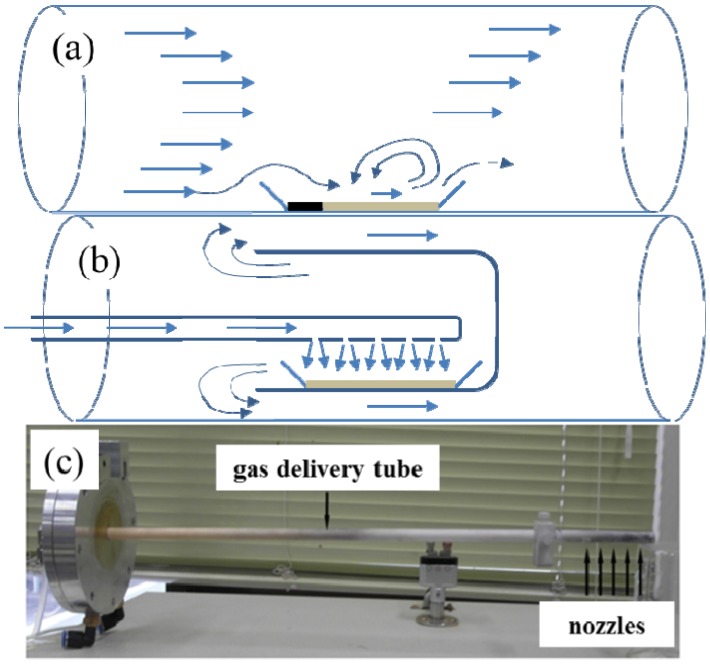
Gas flow schemes for: (**a**) a conventional flow-through reactor; (**b**) a nozzle-type reactor; and (**c**) the actual photo of the nozzle system.

Figure 1a,b shows the gas flow scheme for a conventional flow-through reactor and a nozzle-type reactor, while the actual photo of the nozzle system is shown in Figure 1c.

As shown in Figure 1b,c, the gas-delivering alumina tube, whose end has several small holes, was installed at the center of the cylindrical furnace. The alumina boat (10 mm × 50 mm) containing the precursor powder was located right below the nozzles inside the cap. Before and after the annealing of the precursor powders, the weights of the samples were measured to estimate the BNNTs synthesis in the samples. The specific surface area of each sample was also measured using BET. The synthesis of BN was observed by the X-ray diffraction (XRD, D8 Discover Bruker, Karlsruhe, Germany) patterns, while the growth of NTs was confirmed by scanning electron microscope (SEM, Sirion FEI, Eindhoven, The Netherlands). The inner structure of BNNTs was also observed using a high resolution transmission electron microscope (HRTEM, JEM-2100F JEOL, Tokyo, Japan). The elemental constitution of the samples was analyzed by energy dispersive X-ray (EDX) spectroscopy coupled with SEM and transmission electron microscope (TEM). All experiments were performed using a conventional flow-through reactor and/or crystalline boron-based precursor for comparison purposes.

## 3. Results and Discussion

### 3.1. Preparation of the Precursor for Boron Nitride Nanotubes Synthesis

**Figure 2:**
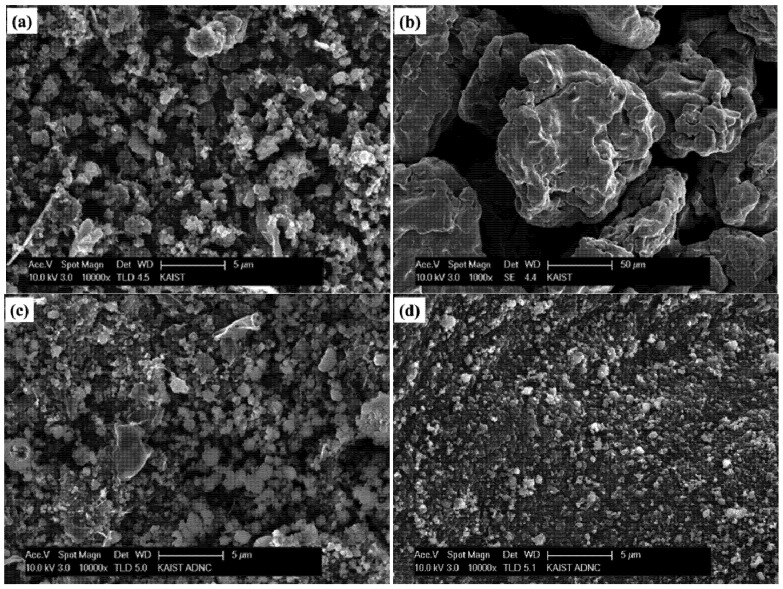
Scanning electron microscope (SEM) images of: (**a**) initial amorphous boron; (**b**) initial Fe; (**c**) the ball-milled amorphous boron and Fe mixture; and (**d**) the supernatant of the ball-milled amorphous boron and Fe mixture dispersed in ethanol.

The images in Figure 2 show the SEM morphologies of initial amorphous boron, initial Fe, the ball-milled amorphous boron and Fe mixture, and the supernatant of the ball-milled amorphous boron and Fe mixture dispersed in ethanol. The particle sizes of amorphous boron ranged from sub-micrometers to several micrometers, while the Fe particles are sized ~100 μm. After ball milling of boron and Fe powder, the size of most particles appears to be reduced to the submicron scale, as shown in Figure 2c, while some large particles, presumably Fe, are still shown in the image. To remove those large Fe particles and their aggregates with boron, a flat magnet was placed below the ethanol beaker for several hours, and the blackish precipitate was separated by decanting the supernatant. The precursor purification efficiency based on the magnet application was measured as 60%–70%. Most ethanol dispersed particles are shown to be submicron scaled, as shown in Figure 2d. The SEM images of the crystalline boron-based precursor can be found in the related literatures [22,34].

**Figure 3:**
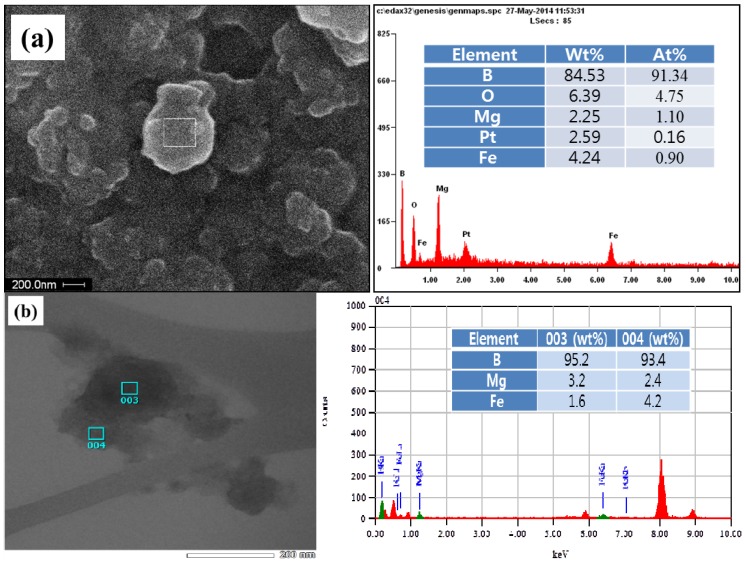
Elemental constitution of B/Fe precursor nanoparticles using: (**a**) SEM; and (**b**) transmission electron microscope (TEM) images coupled with the energy dispersive X-ray (EDX) spectrum.

Figure 3a shows the SEM image coupled with the EDX spectrum of the ball-milled particles. The elemental composition of the arbitrary region in a milled particle indicates ~80 wt% of B and ~6 wt% of Fe. The elements, such as O and Pt, may come from the oxidation of the sample and from the grid coat, respectively, while Mg is pre-existed in the amorphous boron powder. Based on the EDX analysis, it can be found that the precursor nanoparticles fabricated by the ball milling of a mixture of amorphous boron and Fe consist of boron intermixed with Fe. The inner structure of the B/Fe nanoparticles was also observed by the TEM image coupled with EDX spectrum, as shown in Figure 3b. The TEM image of the B/Fe nanoparticles also exhibits the form of boron interdiffused with Fe, and the Fe concentration at each spot is slightly less than that of Figure 3a. This type of B/Fe precursor is clearly different from the precursor fabricated by the ball milling of crystalline boron, which is the Fe nanoparticle encapsulated by disorder-structured boron [22,34]. In these cases, the Fe nanoparticles are originated from an STS-based milling system as an impurity and are coated with amorphous boron (due to the amorphization of crystalline boron). By using amorphous boron mixed with a small amount of Fe, however, the precursor for BNNTs synthesis can be prepared with a much shorter milling time, less than 6 h, while the conventional preparation of the precursor requires a much longer milling time than 24 h and up to 150 h [20,21,22,30,31,32,33], Furthermore, the precursor for the former contains much less Fe impurity than those for the latter, since unwanted Fe particles can be easily removed, as was found earlier.

### 3.2. Characteristics of Synthesized Boron Nitride Nanotubes

#### 3.2.1. Chemical Reaction Dependent on the Gas Flow Scheme

**Figure 4:**
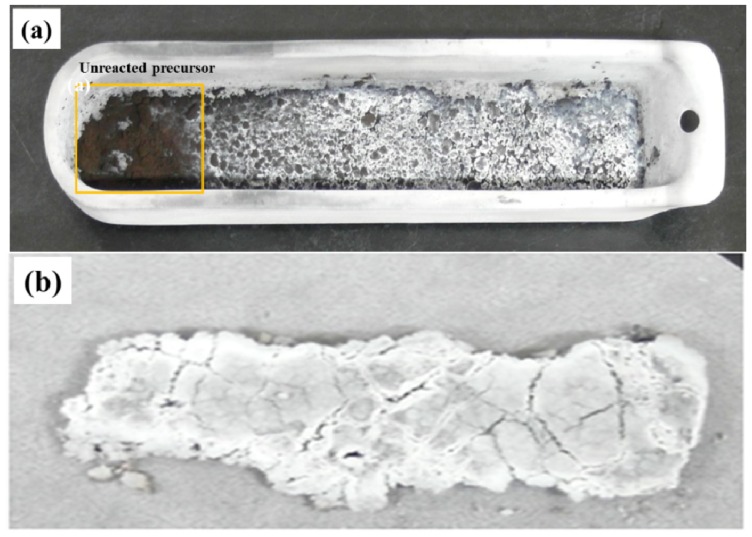
Comparison of the annealed samples synthesized from: (**a**) a conventional flow-through reactor; and (**b**) a nozzle-type reactor.

The synthesis of BNNTs was carried out using an amorphous boron-based precursor with a conventional flow-through reactor and a nozzle-type reactor, respectively. Figure 4 shows the actual photos of the samples after annealing the amorphous boron-based precursor using a conventional flow-through reactor and a nozzle-type reactor. The color of the black precursor changed to a white color, indicating the synthesis of BN, while the synthesis in the nozzle-type reactor appears to be more efficient than the conventional flow-through reactor, according to the color of the samples. As expected from Figure 1a, the front side precursor in the sample boat in the flow-through reactor was not fully reacted, due to the unreached gas flow. On the other hand, the precursor in the nozzle-type reactor could be homogeneously reacted with nitrogen synthesizing BN, due to more efficient gas exposure. Here, it is necessary to verify the synthesized samples if they are BN or something else using the XRD patterns and if they are BNNTs using the SEM/TEM images.

#### 3.2.2. X-ray Diffraction of Annealed Samples

**Figure 5:**
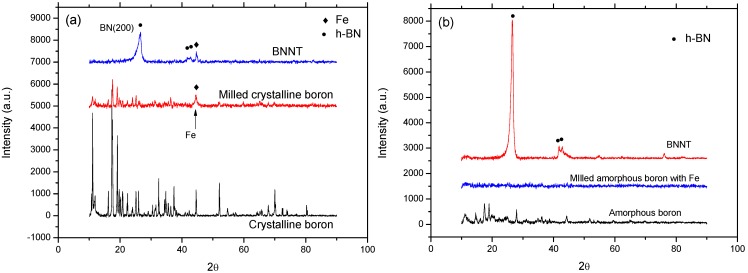
X-ray diffraction (XRD) patterns for annealed samples using the: (**a**) crystalline boron-based precursor; and (**b**) amorphous boron-based precursor with a nozzle-type reactor. BN: boron nitride; andBNNT: boron nitride nanotube.

The XRD patterns shown in Figure 5 are for the annealed samples synthesized from the crystalline boron-based precursor and the amorphous boron-based precursor using a nozzle-type reactor. The XRD patterns in Figure 5a show the initial crystalline boron, ball-milled crystalline boron and annealed samples, respectively, while Figure 5b shows the initial amorphous boron, ball-milled mixture of amorphous boron and Fe (supernatant) and annealed samples, respectively. By comparing the crystallographic status of boron, the roles of the precursor prepared from amorphous boron for the synthesis of BNNTs could be evaluated. According to Figure 5a, the intensity of the peaks for rhombohedral boron (JSPDS No. 80-0322) was reduced after milling, while the peak for cubic Fe (JSPDS No. 87-0721) appears newly in the milled sample as an impurity produced from the milling system. The milling time for this case was set at 12 h for the best result from the previous investigation [22]. The decreased peaks for boron reveal that a large fraction of milled boron loses its crystallinity, and the broad Fe peak at 2θ ~ 45° demonstrates that it is produced as nanosized particles. The synthesis of BN can be confirmed by the strongest (002) peak for hexagonal BN (JDPDS No. 85-1068) shown at 2θ ~ 26° for the annealed sample. This might be strong crystallographic evidence of BNNTs synthesis when considered together with the SEM images.

For amorphous boron case shown in Figure 5b, the relatively weak XRD peaks are shown for initial boron, which may come from the imperfect amorphous status; while the peaks for the milled mixture of amorphous boron and Fe (supernatant) disappeared, demonstrating that the milling of imperfect amorphous boron was able to produce nearly perfect amorphous boron. It is also interesting to notice that the Fe peaks for the ball-milled mixture of amorphous boron and Fe are not observed, due to the limited generation of Fe from the milling system and purification of the Fe particles by the application of the magnet to the ethanol dispersed sample. A small amount of remnant Fe is assumed to only remain in the B/Fe interdiffused nanoparticles, as shown in Figure 3, and can be used as a catalytic agent for BNNTs synthesis. The BN (002) peak for the annealed sample shown in Figure 5b is much stronger than that of Figure 5a, and the peak for Fe is not observed same as the precursor. This demonstrates that the B/Fe interdiffused precursor might be more efficient than the Fe seed nanoparticles encapsulated by amorphous boron for the synthesis of BNNTs, while maintaining the sample purity.

#### 3.2.3. Morphology of Annealed Samples

**Figure 6:**
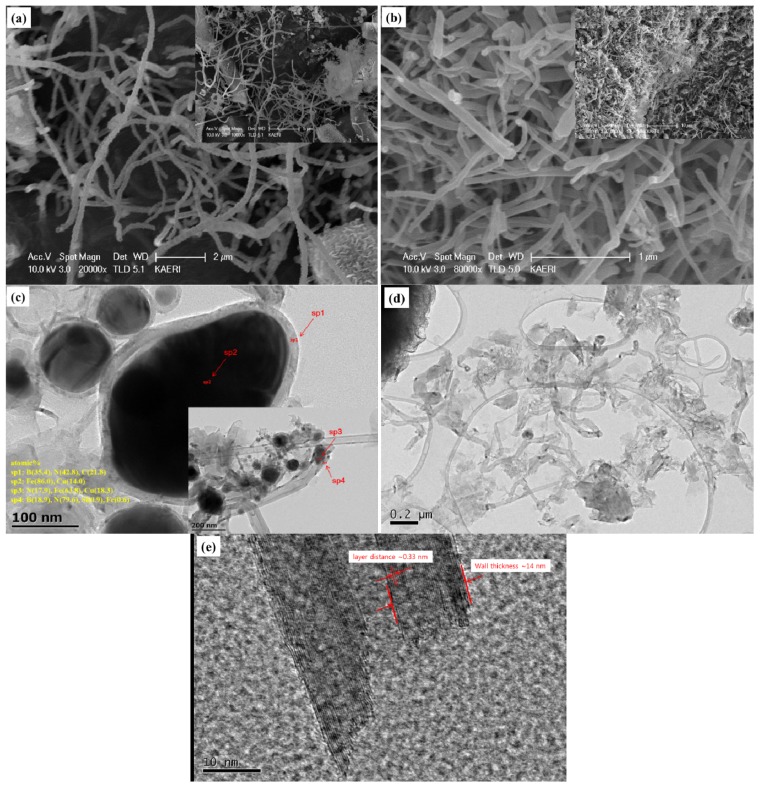
SEM images of BNNTs samples synthesized from the: (**a**) crystalline boron precursor; and (**b**) amorphous boron precursor. TEM images of BNNTs samples synthesized from the: (**c**) crystalline boron precursor; and (**d**,**e**) amorphous boron precursor with a nozzle-type reactor.

Based on the SEM/TEM images of the synthesized BNNTs samples, we were able to find several significant differences in the synthesis of BNNTs between the crystalline boron precursor and amorphous boron precursor. Figure 6 shows the SEM images of BNNTs synthesized from the crystalline boron precursor and amorphous boron precursor, and the TEM images of BNNTs synthesized from the crystalline boron precursor and amorphous boron precursor, respectively.

The diameter and length of BNNTs appear to be varied for both precursors. The NTs from the crystalline boron precursor are relatively longer than those for the amorphous boron precursor, while the diameters for the former are more uniform than the latter. Figure 6c,d shows the inner structure of some immature NTs, which might indicate the initial stage of the NTs growth. From the TEM images, it is interesting to notice that the Fe seed nanoparticles are commonly observed in the sample from the crystalline boron precursor in Figure 6c, while they are hardly observable for the amorphous boron precursor in Figure 6d. The atomic concentrations in the spots, sp2 and sp3, for the seed nanoparticles are 86.0% and 63.8% with the Cu balance (from the grid) and also N (17.9%) for the latter. This agrees well with the literature [22,34] that the seed Fe nanoparticles produced from the crystalline boron are encapsulated by disordered boron synthesizing BN during annealing. The atomic ratio at sp1 is B(35.4%):N(42.8%), and B(18.9%):N(79.6%) for sp4, while N is much richer in the spot, sp4, shown in the inset image in Figure 6c. Unlike Figure 6c, the distinguishable seed nanoparticles are not observed in the TEM image for amorphous boron precursor in Figure 6d. This agrees well with the status of the precursors observed in Figure 3. Furthermore, BNNTs are well crystallized with the multiwalled structure, whose wall thickness is ~14 nm with a layer distance of ~0.33 nm. From this observation, it can be found that the NTs might be also grown from the Fe diffused amorphous boron precursor differently or more efficiently than the previous schemes [22,34]. In addition, it is interesting that most grown and/or premature NTs look to be cylindrically shaped. This agrees well with the previous investigation that the shape of the boron coat on the surface of the seed nanoparticles may determine the type of NTs, cylinders or bamboo. Due to the absence of the irregularly-shaped nano-clusters surrounding the amorphous boron nanoparticle precursor, most NTs are highly probable to grow into the cylindrical type.

### 3.3. Production Yields of Boron Nitride Nanotubes

BNNTs were synthesized under different types of reactors and precursors. Table 1 shows the sample numbers with the assigned experimental schemes and the conditions, and the production yields for each designated sample are estimated in Table 2. By measuring the weight increase of the annealed samples, the degree of BNNTs synthesis was estimated. It was assumed that the weight increase might be solely from the chemical reaction of boron with the supplied nitrogen, synthesizing BN, and no other chemical reactions were expected to form unwanted chemical species based on the XRD patterns of the annealed samples. When the flow-through reactor was used, the weight increase for the samples synthesized from the amorphous boron precursor was higher than ~25% from the crystalline boron precursor. In addition to this enhancement, it was increased ~18% for the nozzle-type reactor when the amorphous boron precursor was used. The specific surface area of each sample was also measured using BET. In fact, the estimation of the quantity and/or yield of BNNTs using the specific surface area of the samples is not possible, since there are so many factors affecting the specific surface area, such as the diameter, length, types of NTs, openness of the end-tip, number of the walls, *etc.* Nevertheless, it might be useful to evaluate the portion of the nanostructured species in the samples by comparing them across samples. The specific surface area (54.6 g/m^2^) for the amorphous boron-based precursor and nozzle-type reactor was increased as much as 2.5-times compared to the crystalline boron-based precursor and flow-through reactor (21.5 g/m^2^). Even though this may not reflect the true sample status, it must be worthwhile to consider the degree of BNNTs synthesis together with the quantitative production yields by the sample weight increase. Under the assumption that the synthesized BN has the shape of the NTs according to the SEM/TEM images, the BNNTs yields based on the weight increase are estimated as 69.7% for the former and 54.1% for the latter, respectively. 

**Table 1:** Experimental schemes and conditions dependent on the reactor and precursor types.

Sample No.	Precursor type	Milling condition	Annealing condition	Reactor type
1	Crystalline boron based	600 rpm, 12 h	1200 °C, 6 h	Conventional flow-through reactor
2	Amorphous boron based			
3				Nozzle-type reactor

**Table 2:** Estimation of BNNTs production yields.

Sample No.	Initial weight of precursor (g)	Weight increase after annealing (g)	BET (m^2^/g)	Estimated BNNTs quantity (g)	Estimated yields of BNNTs (%)
1	1.0	0.44	21.5	0.78	54.1
2	1.0	0.55	40.5	0.97	62.6
3	1.0	0.65	54.6	1.15	69.7

## 4. Conclusions

High production yields of BNNTs were achieved using an amorphous boron-based precursor and a nozzle-type reactor. The precursor for BNNTs synthesis was prepared as the B/Fe interdiffused nanoparticles by the ball milling of a mixture of amorphous boron and Fe with a much reduced milling time. Furthermore, the Fe impurity contained in the precursor after ball milling could be purified easily by the application of a magnet, unlike the crystalline boron-based precursor. The use of the B/Fe precursor is more efficient for increasing the production yield of BNNTs with high purity than the crystalline boron-based precursor. The nozzle-type reactor also contributes to the increase of the BNNTs production yields when compared to the flow-through reactor. The increase of the production yields were estimated quantitatively using the weight gain of the samples after annealing, as well as qualitatively using the BET-specific surface area of each sample. Consequently, the use of the B/Fe interdiffused precursor and a nozzle-type reactor can enhance the production yield considerably with much shorter precursor preparation time and less impurity. The methods presented in this manuscript may offer a closer and faster way to the commercial production of BNNTs.
